# Disulfiram neuropathy: two case reports

**DOI:** 10.1186/s13256-016-0865-z

**Published:** 2016-03-31

**Authors:** Anh Thu Tran, Richard A. Rison, Said R. Beydoun

**Affiliations:** 1 Neuromuscular Division, Department of Neurology, Keck School of Medicine, University of Southern California, 1520 San Pablo Street Suite 3000, Los Angeles, CA 90033 USA; 2 Keck School of Medicine, University of Southern California, Los Angeles County Medical Center; PIH Health Hospital-Whittier; Neurology Consultants Medical Group, 12401 Washington Boulevard, Whittier, CA 90602 USA; 3 Keck School of Medicine, University of Southern California, Los Angeles County Medical Center, 1520 San Pablo Street Suite 3000, Los Angeles, CA 90033 USA

## Abstract

**Background:**

Neuropathy is a rare adverse side effect of disulfiram therapy and is under-recognized. There have been few case reports documenting this side effect.

**Case presentation:**

Two cases of disulfiram peripheral neuropathy are discussed. The first case is that of a 25-year-old Caucasian woman who was exposed to disulfiram therapy for a total of 8 months and developed pain and stiffness that prevented her from walking. The second case is that of a 46-year-old Caucasian woman who developed sudden-onset numbness in her lower extremities with progression to pain. Her symptoms improved over the course of 2 months after cessation of disulfiram therapy. In both cases, symptoms improved after cessation of disulfiram therapy.

**Conclusions:**

Disulfiram neuropathy occurs in persons with a history of chronic alcohol use. It is under-recognized and often attributed to alcoholic neuropathy given its comorbidity with alcoholic neuropathy. A greater understanding of this side effect may reduce neurologic complications related to disulfiram neuropathy and aid in early withdrawal of this offending agent.

## Background

Disulfiram is typically used as a second-line agent for treating chronic alcoholism. Alcohol is metabolized by alcohol dehydrogenase to acetaldehyde, which is then broken down by aldehyde dehydrogenase to acetic acid and excreted. Disulfiram’s main mechanism of action is to inhibit aldehyde dehydrogenase, thereby increasing serum acetaldehyde levels. When alcohol is taken in conjunction with disulfiram, the drug interaction results in high levels of acetaldehyde. This produces an unpleasant physiologic reaction, causing patients to have “hangover” symptoms such as nausea, palpitations, flushing, vertigo, and chest pain. Adverse reactions have been documented in both the central and peripheral nervous systems. In the central nervous system, adverse reactions include drowsiness, headache, fatigue, polyneuritis, and psychosis. Adverse reactions in the peripheral nervous system include peripheral neuritis and peripheral neuropathy. Several authors have suggested that this physiologic reaction is dose-dependent and related to an accumulation of carbon disulfide, which is a by-product of the metabolism of disulfiram in the liver [1]. Disulfiram neuropathy has been described as a distal axonopathy related to the “dying-back” effect of axonal degeneration [2–4]. The frequency of these adverse reactions has not been defined. A brief literature review showed that the adverse reaction of peripheral neuropathy is uncommon.

## Case presentation

### Case 1

Our first patient was a 25-year-old Caucasian woman who was referred to our tertiary neuromuscular clinic for a second opinion regarding her neuropathy. She had a history of heavy alcohol use from ages 18 to 23, during which she consumed as many as eight to ten drinks per day. She first had sensory symptoms at age 22, described as numbness of her feet as if they were “falling asleep.” She initially did not pay attention to the symptoms. Gradually, her symptoms progressed and included burning, electric shock sensation, and allodynia to objects including bed sheets, socks, and the floor when walking barefoot. She was placed on disulfiram therapy (500 mg orally every morning) for 6 months, then discontinued for several months before it was reinstituted for an additional 2 months at the same dose. By the eighth month of therapy, she was unable to walk owing to severe symptoms of pain and stiffness in her legs. She also noted symptoms in her hands. She stopped taking the disulfiram and was able to walk within a few weeks, but she continued to have pain and numbness.

She had seen a community neurologist prior to seeking a second opinion in our clinic. Laboratory studies completed previously showed normal levels of HbA1c, vitamins B1 and B12, folate, Sjögren syndrome antigen A and B antibodies, antinuclear antibody, antineutrophil cytoplasmic antibodies, and antigliadin antibodies. She had monoclonal gammopathy, and results of a sensorimotor neuropathy antibody panel were within normal limits. Pertinent examination findings showed that she had normal motor function except for decreased bulk in her extensor digitorum brevis. A sensory examination showed a gradient distribution decrease in light touch, temperature, and pinprick distally, along with decreased vibration and position sense at her toes. Her gait and reflexes were normal. Electrodiagnostic studies showed a severe length-dependent motor neuropathy, as evidenced by a significantly decreased amplitude in the compound motor action potential (CMAP) in her lower extremities with a preserved CMAP in her upper extremities. There was sparing of her large fiber sensory nerves, as evidenced by normal sensory nerve action potentials (SNAPs). Both right and left posterior tibial CMAP waveforms showed temporal dispersion as well as prolonged duration. Electromyography (EMG) was performed on selected muscles in her bilateral lower extremities and revealed 2+ fibrillation potentials and positive sharp waves in her abductor hallucis muscles. Also seen were polyphasia, increased amplitude and increased duration with decreased recruitment, and increased firing rate to most muscles sampled.

### Case 2

Our second patient was a 46-year-old Caucasian woman who was being followed by a community neurologist, to whom she reported sudden onset bilateral numbness of her feet when walking. She had progressively worsening symptoms, including freezing/burning pain, and allodynia to bed sheets and when walking barefoot. She was initially referred to our tertiary neuromuscular clinic; however, she was admitted to the Keck Medical Hospital owing to the acutely progressive nature of her symptoms. During her hospital stay she had an unremarkable workup, including lumbar puncture, cervical and thoracic MRI, as well as chest, abdominal, and pelvic CT. Results from an additional laboratory workup, including testing for vitamin B12, folate, TSH, rapid plasma reagin, heavy metals, HbA1c, and homocysteine, and a serum electrophoresis were normal.

She presented to our clinic a month after being discharged from the hospital. She reported a 3-month history of sudden-onset numbness in both feet that had worsened and which she described as a frostbite sensation of freezing and burning. She had allodynia to bed sheets and walking barefoot that she described as a sensation of “walking on pegs.” Her social history showed heavy alcohol use of one bottle of wine daily. She had taken disulfiram at a dose of 500 mg daily but had stopped the medication a couple of weeks prior to her hospital admission when she developed symptoms of neuropathy. Since her discharge from hospital, she reported that she was no longer feeling pain and that the frostbite sensation had improved. Pertinent findings included normal results from a motor examination except for weaker bilateral ankle dorsiflexion. A sensory examination showed distal changes including absent position sense at the toes, significantly decreased vibration at the toes, and absent light touch and pinprick to the distal 5 cm of her feet. Electrodiagnostic testing was performed several weeks after her initial clinic visit and a nerve conduction study revealed severe motor axonal neuropathy in her lower extremity nerves with length-dependent characteristics. Her bilateral peroneal CMAPs were significantly decreased. By contrast, her upper extremity CMAP parameters were normal. There was sparing of her large fiber sensory nerves as evidenced by normal SNAPs. EMG showed 1+ positive sharp waves and fibrillation potentials in most of the sampled muscles of her lower extremities. There were slight to moderate increases in the amplitudes of the motor unit potentials as well as increased polyphasia.

Please see Tables 1 and 2 for electrodiagnostic data for both patients.

**Table 1:** Summary of electrodiagnostic data – motor studies

Nerve (normal values)	Case 1		Case 2	
	Right	Left	Right	Left
Tibial				
Lat (m/s) (<5.9)	5.3	3.6	6.6	4.6
Amp (mV) (>3)	0.8	0.9	0.3	0.0
CV (m/s) (>39)	34.3	39	52.5	N/A
Peroneal				
Lat (m/s) (<6.5)	4.7	5.0	3.7	8.5
Amp (mV) (>2.1)	1.8	0.9	0.1	0.1
CV (m/s) (>39)	45.1	46.8	34.9	38.7
Median				
Lat (m/s) (<4.5)	N/A	2.3	N/A	4.0
Amp (mV) (>4.9)	N/A	12.1	N/A	7.2
CV (m/s) (>48)	N/A	54.2	N/A	54.3
Ulnar				
Lat (m/s) (<4.5)	N/A	2.3	N/A	2.7
Amp (mV) (>4.9)	N/A	11.3	N/A	7.6
CV (m/s) (>48)	N/A	55.1	N/A	58.3

*CV* conduction velocity, *Lat* latency, *Amp* amplitude, *m* meter, *s* second, *mV* millivolt

**Table 2:** Summary of electrodiagnostic data – sensory studies

Nerve (normal values)	Case 1		Case 2	
	Right	Left	Right	Left
Sural				
Lat (m/s) (<4.4)	3.2	3.8	3.6	3.4
Amp (mV) (>5.9)	10.24	11.33	4.25	8.35
CV (m/s) (>39)	43.7	45.4	48.2	48.2
Median				
Lat (m/s) (<3.8)	N/A	3.0	N/A	3.6
Amp (mV) (>19)	N/A	44.64	N/A	57.09
CV (m/s) (>49)	N/A	59.8	N/A	47.2
Ulnar				
Lat (m/s) (<3.7)	N/A	3.0	N/A	3.2
Amp (mV) (>16)	N/A	25.92	N/A	28.38
CV (m/s) (>49)	N/A	58.3	N/A	51.8

*CV* conduction velocity, *Lat* latency, *Amp* amplitude, *m* meter, *s* second, *mV* millivolt

### Discussion

Distal axonopathy is considered the hallmark of disulfiram toxicity and was evident in these two cases. Axonopathy is demonstrated by the low CMAPs with relatively normal motor conduction velocities. The length-dependent nature of the disulfiram neuropathy was demonstrated by the severely decreased CMAP in the lower extremities while the upper extremities were spared. Demyelination was not supported by the results of either laboratory studies or electrodiagnostic testing. Studies suggest the causal agent of disulfiram neuropathy is carbon disulfide, which is a by-product of disulfiram metabolization [1]. Ansbacher *et al.* reported a case in which a sural nerve biopsy demonstrated neurofilamentous distal axonopathy and cited carbon disulfide as the responsible agent. Clinically, disulfiram neuropathy and alcoholic neuropathy can be difficult to distinguish. Some observations that can help distinguish the two include a history of onset that occurs in a matter of weeks in disulfiram neuropathy as opposed to an insidious course over months in alcoholic neuropathy. Additionally, the progression is faster in disulfiram neuropathy [5]. Symptoms of both types of neuropathy can be similar, such as symmetrical distribution, worse severity distally, and depression of ankle jerk reflexes. In contrast to alcohol neuropathy, disulfiram neuropathy results in muscle tenderness as well as disturbances in sweating of distal limbs [6]. In our two cases, our patients presented with distal lower extremity symptoms of predominantly painful sensory complaints. Our patient in case 1 had pre-existing symptoms prior to disulfiram therapy, but experienced an acute worsening of symptoms following disulfiram therapy. Our patient in case 2 had lower extremity symptoms of sudden onset and a temporal relationship with disulfiram therapy. Symptoms in both patients progressed at a faster rate than typically observed with alcoholic neuropathy at a time when both were abstaining from alcohol. Clinically, symptoms improved in both patients after cessation of disulfiram; most notably, our patient in case 2 was free of pain 2 months after stopping disulfiram. Electrodiagnostic tests in both patients demonstrated severe length-dependent axonopathy with low distal CMAPs in their lower extremities but normal CMAPs in their upper extremities. EMG studies showed both active denervation, as evidenced by fibrillation and positive sharp waves, as well as chronic denervation, demonstrated by increased amplitude and polyphasia. Additionally, although the primary complaint was sensory in both patients, their SNAPs were spared. This suggests the involvement of small fiber sensory nerves but the sparing of large fiber sensory nerves. This is in contrast to prior literature that report large fiber involvement in disulfiram neuropathy [5, 6]. Unfortunately, both of our patients declined repeat electrodiagnostic testing and hence it was not possible to document a correlation between clinical and electrophysiological findings. Although not routinely performed in the evaluation of disulfiram neuropathy, perhaps sensory nerve biopsies would help elucidate any such correlation.

## Conclusions

Disulfiram neuropathy occurs in patients who have a history of chronic alcohol use. The frequency with which it occurs is difficult to assess, though one quoted figure is 1:15,000 [5, 7]. The challenge in determining disulfiram as the causative agent is that concomitant alcohol-related neuropathy is often a confounding factor; thus, disulfiram neuropathy is likely under-diagnosed and attributed to alcohol neuropathy. Often, clinicians must rely on the timing of neuropathic complaints with respect to disulfiram administration. As with case 1, our patient had sensory complaints prior to starting disulfiram therapy; however, she experienced acute worsening of her symptoms after initiating therapy at a time when she was abstaining from alcohol. Additionally, her symptoms improved with cessation of disulfiram. In case 2, our patient’s neuropathic symptoms clearly developed after she started disulfiram therapy. Her symptoms improved over the course of 2 months after the cessation of therapy. Because the underlying neuropathy due to alcohol is a confounder, we propose further study of the potential effects of disulfiram on peripheral nerves. One method to study this would be to perform electrodiagnostic studies prior to initiating disulfiram therapy to obtain a baseline. Neuropathy and electrodiagnostic findings of decreased CMAP to indicate axonopathy after disulfiram therapy can then be attributed to disulfiram with greater confidence, given that patients will have abstained from alcohol during disulfiram therapy. Another useful study may be to perform a sensory nerve biopsy to determine if small fiber sensory nerves are involved.

## Consent

Written informed consent was obtained from the patients for publication of this case report. A copy of the written consent is available for review by the Editor-in-Chief of this journal.
